# Leaf Area Calculation Models for Vines Based on Foliar Descriptors

**DOI:** 10.3390/plants10112453

**Published:** 2021-11-13

**Authors:** Florin Sala, Alin Dobrei, Mihai Valentin Herbei

**Affiliations:** 1 Department–Soil Sciences, Compartment of Soil Science and Plant Nutrition, Banat University of Agricultural Sciences and Veterinary Medicine “King Michael I of Romania”, 300645 Timisoara, Romania; florin_sala@usab-tm.ro; 2 Department–Horticulture, Compartment of Viticulture, Banat University of Agricultural Sciences and Veterinary Medicine “King Michael I of Romania”, 300645 Timisoara, Romania; alin1969tmro@yahoo.com; 3 Department–Sustainable Development and Environmental Engineering, Compartment Remote Sensing and GIS, Banat University of Agricultural Sciences and Veterinary Medicine “King Michael I of Romania”, 300645 Timisoara, Romania

**Keywords:** foliar descriptors, leaf area, models, vine leaves

## Abstract

In the case of foliar area studies on vines, with a large number of determinations, a simple, fast, sufficiently accurate and low-cost method is very useful. The typology of leaves on the vine is complex, characterized by several descriptive parameters: median rib; secondary venations of the first and second order; angles between the median rib and the secondary venations; sinuses; length and width of the leaf. The present study aimed to evaluate models for calculating the leaf area based on descriptive parameters and KA (KA as the surface constant used to calculate the leaf area) for six vine cultivars, ‘Cabernet Sauvignon’ (CS), ‘Muscat Iantarnîi’ (MI), ‘Muscat Ottonel’ (MO), ‘Chasselas’ (Ch), ‘Victoria’ (Vi) and ‘Muscat Hamburg’ (MH). The determined KA surface constants had subunit values (0.91 to 0.97), except for the cultivars ‘Muscat Iantarnîi’ and ‘Muscat Ottonel’ where the surface constant KA_2_ (in relation to the second-order secondary venations) had supraunitary values (1.07 and 1.08, respectively). The determination of the leaf area was possible under different conditions of statistical accuracy (R^2^ = 0.477, *p* = 0.0119, up to R^2^ = 0.988, *p* < 0.001) in relation to the variety and parametric descriptors considered. The models obtained from the regression analysis facilitated a more reliable prediction of the leaf area based on the elements on the left side of the leaf, in relation to the median rib, compared to those on the right. The accuracy of the results was checked on the basis of minimum error (ME) and confirmed by parameters R^2^, *p* and RMSE.

## 1. Introduction

Foliar parameters are integral elements of the leaves, geometry, found in a certain proportionality with the leaf as a whole, and can be used to evaluate the leaf area, the indices dependent on the leaf area, as well as to study physiological, ecological and agricultural nature of plants [1,2,3,4]. Anatomical elements and descriptive parameters of the leaf lamina were used in the study and ampelographic characterization of genotypes in vines and in the evaluation of ecological plasticity in relation to certain environmental factors [5,6,7,8,9,10].

Some studies have evaluated changes at the molecular, cellular and topological levels of the leaves in relation to the plasticity of the respective genotypes [11,12]. Various other foliar studies have focused on the interception of solar energy [13], photosynthetic rate [14,15,16], nutritional status [17,18,19], water utilization ratio in relation to production [20,21], the peculiarities of growth and development of the vine [22,23], fruiting and production quality [24,25,26,27,28,29], quality of vines for human nutrition and phytopharmaceutical products [30,31], degree of attack of diseases and pests [32], the relationship of the vine with environmental factors and the reaction to stress condition [33,34,35,36,37,38].

Data on leaf parameters and in particular the leaf area, and indices targeting the leaf area (specific leaf weight—SLW, leaf area index—LAI, net assimilation rate—NAR, etc.) were used in assessing the relation of the vine with the ecological and technological factors [20,39]. The leaf area is highly correlated with leaf indices [40,41,42], with canopy cover [41,43,44,45], by the fraction of light intercepted by the canopy [46,47,48] and finally with crop coefficient Kc [49,50].

Descriptive parameters of the leaf lamina were important in the computerized reconstruction of leaves in different types and varieties of vines [51], with practical importance for the realization of study models and some specific foliar fingerprints. Some studies have focused on the seasonal cycle of growth and development in several varieties and varieties of vines, which is why it has been important to analyze the leaves in their dynamics [52,53]. The accurate determination of leaf parameters and leaf area (LA) is a key issue in crop growth analysis [54], as simple regression models relating LA and crop growth rate are commonly used to estimate crop yield [55].

Methods that are non-destructive, fast, low-cost and sufficiently accurate for determining leaf area are of interest due to their high efficiency in vine physiology and technology studies, especially in terms of leaf surface dynamics in relation to various factors.

The present study analyzed the leaf area of six vine cultivars in order to determine the leaf area by means of models developed based on the parameters of leaf lamina and KA surface constants specific to each cultivar.

## 2. Results

The vine leaf has a special typology and complexity that is characterized by a series of descriptive elements: the median rib, secondary venations of the 1st and 2nd order, lobes, sinuses, and angles between the median and secondary venations. Based on these descriptive elements and the general considerations presented, the present study aimed to evaluate some models of leaf surface calculation based on descriptive elements, KA surface constants and regression analysis. The surface constants are specific to each variety by leaf typologies, and the dimensions of the descriptive parameters of the leaves can be obtained with high accuracy by measurements (±0.5 mm). The study aimed to evaluate through a comparative analysis where foliar parameters and calculation method most easily facilitate the determination with high precision of the leaf area, to be promoted in the case of studies targeting a large number of leaf area determinations in the vine of life.

Measurements were made for each leaf on the median rib, the secondary venations of order 1 and 2, the distances between the terminations of the venations of order 1 and those of order 2, on the distances from the base of the sinuses to the base of the median rib, and, respectively, on angles α and β, the results being shown in Table 1 and Table 2. At the same time, each leaf was scanned, resulting in the scanned leaf area (SLA) with high-precision (99.95–100.00), considered as a reference area for further comparisons with measured leaf area (MLA) in the study.

Based on the leaf sizes obtained by measurement, the surface constants KA_1_ and KA_2_ were determined, the results being presented in Table 3. The optimal values for the surface constants were considered under the conditions of the minimum error between MLA and SLA (considered as reference) and of the statistical parameter RMSE.

The determined KA surface constants had subunitary values, except for the cultivars ‘Muscat Iantarnîi’ and ‘Muscat Ottonel’, where the KA_2_ surface constant had supraunitary values. Based on the KA values determined for each variety, the values of the leaf areas were calculated with high precision. From the comparative analysis of the obtained results, it was found that the leaf area was determined with greater precision based on the secondary venations of order 2 and KA_2_, except for the cultivars ‘Muscat Iantarnîi’ and ‘Victoria’.

The studied vine varieties presented a distinct foliar typology, genetically well-defined and characterized ampelographically [56], which was reflected in the clear differentiation of the results regarding the morphological and foliar surface elements. According to the values of the obtained leaf areas and the leaf descriptors [57], the leaves of the cultivars ‘Cabernet Sauvignon’, ‘Muscat Iantarnîi’, ‘Muscat Ottonel’ and ‘Chasselas’ were classified in class 8.5-Mesophyll (4500–18,225 mm^2^) and the cultivars ‘Victoria’ and ‘Muscat Hamburg’ in class 8.6-Macrophyll (18,225–164,025 mm^2^).

In the case of studies of vegetation dynamics in vines, with a high number of determinations, a simple, fast, sufficiently accurate and low-cost method is very useful. Based on the correlations identified between the values of the descriptive parameters of the leaf lamina, the prediction of leaf surfaces was evaluated only based on a known element at the level of the leaf lamina.

For this purpose, the regression analysis was used for each of the descriptive elements studied, the equations describing the prediction relations and statistical accuracy parameters being presented in Table 4. In the case of estimating the leaf area based on the angles α and β, no statistical certainty was registered, and the results were not taken into account.

The regression analysis based on descriptive parameters of the leaves led to the Equations (1)–(11), which predict the leaf area (PLA) in statistical accuracy conditions. Based on the values of the RMSE parameter and the correlation coefficient R^2^, it was found that the elements on the left side of the median ribs (VL1, VL2, DSL1 and DSL2) facilitated the more accurate prediction of the leaf area compared to those on the right (VR1, VR2, DSR1 and DSR2). Such findings have not been found in the literature.

The analysis of statistical accuracy parameters (R^2^, RMSE) found that the descriptive elements on the left side of the leaves facilitated a higher accuracy in determining the leaf area compared to the homologous elements on the right side (most likely due to leaf asymmetry, but the level of asymmetry has not been assessed), which recommends their use for calculating the leaf area in cultivars studied, when using only one known element of the leaf.

## 3. Discussion

Different methods can be used to determine the leaf area, classified into two broad categories, destructive and nondestructive, and direct and indirect, respectively [58]. Kvet and Marshall [59] concluded that the most appropriate method is the one in relation to the volume of plant material to be determined, the required accuracy, the time interval, the staff involved and the allocated costs, the planimetric determination or by scanning, providing the highest accuracy.

Direct methods for determining leaf area are based on measurements of leaf size and can be destructive, with greater accuracy [60,61,62], or non-destructive with portable devices or based on leaf size [60,63,64,65,66].

Destructive methods are generally more accurate but are more laborious, costly in terms of time, equipment and personnel. The simplest method is based on measuring the leaf area by planimetry or graph paper [58,67]. The gravimetric method, which is sufficiently accurate, is based on the exact determination of the weight of known surfaces (rectangular or circular) in a leaf to obtain a fit line, and the subsequent correlation with the weight of the leaves of interest to find the leaf surface [68]. However, this method is highly dependent on the cultivar, vegetation stages, plant age, leaf density, nutritional status and especially the hydration status of the leaves [69,70,71,72]. In some studies, the determination of leaf area was performed by combined non-destructive (scanning with portable devices) and destructive (gravimetric) methods [73,74]. Increasingly promoted are non-destructive methods that facilitate the repetitive study of leaves in the dynamics of growth and development processes in field conditions, for which portable scanners [17,53], imaging-based techniques [75,76,77], simple measurement methods based on leaf size [64,78,79] or mathematical and statistical models developed based on leaf size are used [80,81,82]. A number of other techniques have been proposed for estimating the leaf area in vines, based on indirect methods, such as imaging by measuring light extinction through the canopy [61,65,83,84,85,86], remote sensed imagery [87,88], ultrasonic-based method [89], remote sensing combined to Smart-App [90], or based on 3D point clouds resulted from UAV imagery [91]. In the case of such methods, a number of climatic, atmospheric parameters, or other external factors, can influence the accuracy according to which the leaf area is determined [92,93,94]. At the same time, these methods are very expensive because they require specialized equipment and certain calibration works, but they offer the possibility determining the leaf area and derived indices (leaf area index—LAI, leaf area duration—LAD, net assimilation rate—NAR, specific leaf area—SLA, specific leaf weight—SLW) over relatively large areas [84,95,96,97]. 

Indirect methods were used to determine the leaf area, canopy structure and leaf area index (LAI) in relation to different crops, climatic conditions, cropping systems and working techniques [84,98]. Williams and Ayards [20] found that the leaf area is in a linear relationship with LAI indices, water consumption and crop coefficient (Kc) in statistical accuracy conditions (R^2^ = 0.89). Other research found the linearity relationship of the leaf surface with Kc and LAI [99]. The direct, non-destructive, in situ methods that use leaves dimensional parameters, relatively easy to measure, to leaf area estimation, are simple, fast, sufficiently accurate, with affordable costs and tools [58,100]. They are based on leaf length (L), maximum width (W), petiole length (Lp), leaf length x maximum width (LW), the square of the length (L2), the square of the width (W2) or some combination of these variables [101,102,103,104]. To determine the leaf area based on leaf size (L,W) in some studies, correction factors were used [104,105,106] or surface constants Kl or Kf [107] for the gravimetric method, which brought an extra precision to the calculation of the leaf area. 

The estimation of the leaf area by using the leaf dimensions based on mathematical models was of interest due to its high speed and accuracy, certain parameters derived from statistical safety in calculations (R^2^, *p*, RMSE) and the ability to estimate the accuracy level for subsequent comparisons with other results. However, when certain mathematical models were used to estimate leaf area in different crops, few models were used in vines to calculate leaf area [108]. The complexity of the vine leaf has led some models to develop based on the median vein [92,109], of lateral nerves of the first or second order [110,111,112], or based on the maximum length and width of the leaves [60,63,64,113]. To minimize errors, different leaf samples were proposed, such as number and position on the rope, then extrapolated to plant-level data, if necessary. Thus, Carbonneau [111] proposed measuring one leaf sample in each group of four contiguous leaves without losing accuracy, while Barbagallo et al. [114] proposed an empirical model to estimate primary leaf area per shoot based only on the measurement of three leaves: the largest leaf, the apical leaf and an intermediate leaf. These methods greatly reduce the workload if it is necessary to determine the leaf area for the whole plant and for many variants. Mabrouk and Carbonneau [115] proposed a model for determining the entire leaf area per shoot in the Merlot variety, based on the correlation between the total leaf area and the length of the primary and lateral shoots. 

Good estimations of leaf area were found by using a model based on leaves in selected positions on the shoot [114]. Subsequent studies have shown that shoot length, however, is not always closely correlated with leaf area, especially for primary shoots [112,116]. Barbagallo et al. [117] found that cultivar and climatic and cultural factors affected linear and/or multiple regressions (using shoot length and leaf number as independent variables) to such an extent that it could not be used to accurately estimate leaf area per shoot. Another empirical model for estimating the leaf area per shoot has been proposed by Lopes and Pinto [112], which includes four variables: shoot length, number of primary leaves and the area of the largest and smallest leaves. Beslic et al. [118] considered that the method used depends on the cultivar and its leaf characteristics, such as shape, number of lobes, shape of sinuses, etc., and it always assumes the use of a large sample of leaves in order to produce the best prediction. Di Lorenzo et al. [119] found high correlations between shoot length and leaf area, and high correlations are also reported by Lopes and Pinto [80] for varieties ‘Aragonez’, ‘Cabernet Sauvignon’, ‘Touriga Nacional’, ‘Jean’ and ‘Combined’. Complex, multi-variable models [112] provide greater accuracy but require more determination, while simpler methods have a higher margin of error. Based on the results obtained at cv. Blaufrankisch (*Vitis vinifera* L.), Beslic et al. [118] have considered that the original method proposed by Lopes and Pinto [112] is advantageous when it is difficult to determine the largest and the smallest leaf on a lateral shoot, as is the case with cultivars that have numerous and vigorous lateral shoots (which is not the case in cv. Blaufrankisch). Some studies require a large volume of determinations to find the leaf surface in dynamics or on the stem (LA per vine) and in the case of several variants [23,53,120]. 

Numerous studies have reported high accuracy in determining the leaf area in vines based on elements measured at the leaf level. Manivel and Weaver [121] found a high correlation between the length of vine leaves (‘Grenache’ cultivar) and their area (R^2^ = 0.91). Carbonneau [111] and Carbonneau and Mabrouck [122] proposed a method using a number of linear parameters to estimate leaf area (LA). The best results were obtained by adding the lengths of the two main lateral veins. The coefficient of determination was 0.95 when 30% of the leaves on one stem were measured. Lopes and Pinto [112], when analyzing four grapevine cultivars (Fernão Pires, Vital, Touriga National, Periquita), they have obtained the predicted leaf area (PLA) under conditions of higher statistical accuracy when using first-order secondary venations compared to the median rib (the assessment being made on the basis of R^2^). Montero et al. [108] determined the leaf area of the vine, ‘Cencibel’ cultivar, based on leaf size (leaf length and maximum width) obtained by simple regression analysis prediction relations with high accuracy (R^2^ = 0.987 to 0.998). When they used maximum width (W), leaf length (L), petiole length (Lp) and dry weight of leaves (DML) as single variables in the regression equations were not as closely associated with total leaf area, although their R^2^ values were also highly significant. Gutierrez and Lavín [79] determined the leaf area of the vine, Chardonnay variety, based on maximum length × maximum width for the shoot leaves and length between leaf apex and petiolar point × width between points of the superior lobules for the leaves of lateral shoots yielded the best linear mathematical indicators. Based on the determined foliar parameters, they obtained prediction relations of the leaf area with different accuracy levels (estimated based on the coefficient R^2^), which suggests the differentiated contribution of the descriptive parameters of the leaves to the calculation of the leaf area and the need to know and choose those anatomical elements of the leaf that provide the greatest certainty in the calculation/prediction of the leaf area. High values for LA prediction based on median veins and maximum leaf width in two vine varieties (Niagara and DeChaunac) were also reported [113]. The accuracy and safety of the predictions were higher when based on the maximum width of the leaves than on their length. Tsialtas et al. [123] obtained high accuracy in predicting leaf area in the variety Cabernet Sauvignon (R^2^ = 0.97). Similar results were also reported by Beslic et al. [81] to estimate leaf area in cv. Blaufrankisch.

Karim et al. [82] used linear regression models to estimate the leaf area of *Manihot esculenta* in parallel with gravimetric methods based on fresh and dry matter. They concluded that regression models obtained showed linear relationships when actual leaf area plotted against predicted leaf area of another one hundred leaves from different samples and that this confirmed accuracy of the developed models. Moreover, model selection indices had a high predictive ability (high R^2^) with minimum error (low mean square error and percentage deviation). The selected models appeared accurate and rapid but unsophisticated, and they can be used for the estimation of LA in both destructive and non-destructive means in the Philippine Morphotype of Cassava.

Zufferey et al. [124], based on the length of each leaf lamina’s two secondary lateral veins (‘Chasselas’, clone 14/33-4, rootstock 3309 C) and some allometric equations, obtained the leaf surface with statistically higher certainty in the case of secondary nerves based on R^2^. Wang et al. [125] have performed geometric modeling based on B-spline for the study of leaves at Liriodendron. Tomaszewski and Górzkowska [126] have analyzed comparatively the variation of the shape of the leaves in fresh and dry states. Wen et al. [127] have used a multi-scale remashing method for leaf modeling.

In the case of the present study performed on six grape cultivars, the values of the R^2^ coefficient for the prediction relations of the leaf area PLA had high values in the case of LA prediction based on MR, VL1, VL2, VR2 and DV2 (R^2^ = 0.917 to 0.997) and reduced values in the case of prediction based on DSS1 and DSR1. Based on the leaf parameters MR and DV1 or DV2, four cultivars (‘Cabernet Sauvignon’, ‘Chasslas’, ‘Muscat Hamburg’, ‘Muscat Ottonel’) have recorded a higher accuracy and safety prediction of the leaf area based on the secondary venations of order 2 (MR·DV2·KA_2_), and in two cultivars (‘Muscat Iantarnîi’ and ‘Victoria’), a better prediction was obtained based on the first-order venations (MR·DV1·KA_1_). Based on the models obtained from the regression analysis, the elements on the left side of the leaf, in relation to the median rib, facilitated a more reliable prediction of the leaf area compared to those on the right. The reliability of the results was checked on the basis of minimum error (ME) and confirmed by R^2^, *p* and RMSE parameters.

## 4. Materials and Methods

### 4.1. Biological Material

**Figure 1:**
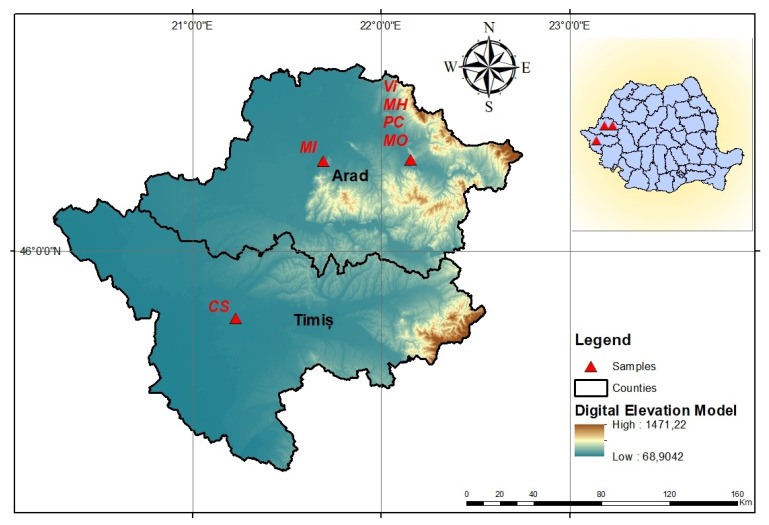
Cultivation area of the studied vine cultivars and leaf sampling locations, Arad and Timiș counties, Romania. CS—‘Cabernet Sauvignon’, MI—‘Muscat Iantarnîi’, MO—‘Muscat Ottonel’, Ch—‘Chasselas’, Vi—‘Victoria’ and MH—‘Muscat Hamburg’. The map was made by the authors using ArcGIS software [128] and their own data.

The study on the determination of leaf area based on descriptive parameters of leaves and KA surface constants was performed on six grape cultivars with different leaf typologies: ‘Cabernet Sauvignon’, ‘Muscat Iantarnîi’, ‘Muscat Ottonel’, ‘Chasselas’, ‘Victoria’ and ‘Muscat Hamburg’. The studied vine cultivars are cultivated in Arad and Timis counties, Romania, Figure 1.

### 4.2. Leaf Sampling

To determine the leaf area by scanning and based on the descriptive elements of the leaf lamina, 30 leaves from each variety were harvested and analyzed. The leaves were harvested in the grain-forming phenophase, BBCH 73–75 stage, and Principal growth stage 7: Development of fruits [129] from the main shoot, in the area of internodes 9–11, considered as typical leaves for characterization of grape cultivars. The leaves were immediately placed in plastic bags in the refrigerator and then transported to the laboratory for determination.

### 4.3. Measurement of Leaf Descriptive Parameters

**Figure 2:**
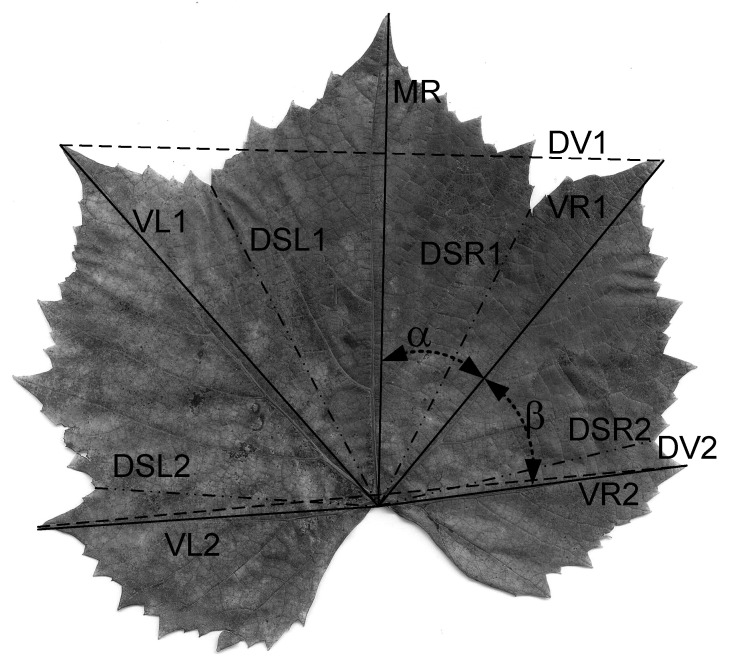
Descriptive parameters determined at the level of the lamina of the vines.

At the level of the leaf lamina, specific descriptive parameters were determined for the vine, Figure 2: Median rib—Midrib (MR); left venation of order I (VL1); right venation of order I (VR1); distance at the end of the venations VL1-VR1 (DV1); second-order left venation (VL2); second-order right venation (VR2); distance at the end of the venations VL2-VR2 (DV2); sinus base distance 1 left to lamina base (DSL1); sinus base distance 1 right to lamina base (DSR1); sinus base distance 2 left to lamina base (DSL2); sinus base distance 2 right to lamina base (DSR2); the angle α between the median rib (MR) and the right venation of the first order (VR1); the angle β between the first-order straight venation (VR1) and the second-order straight venation (VR2).

The measurement of the length of the determined elements was done using a ruler, with an accuracy of ±0.5 mm. The determination of the angles α and β was done using the ImageJ software [130]. Based on the values of the obtained leaf areas and the leaf descriptors [57], the classification of the cultivars studied by leaf size classes was performed.

### 4.4. Determination of Leaf Area

The leaf area was determined for each leaf by scanning with ImageJ software (National Institutes of Health, USA [130]) (scanned leaf area—SLA). The scan was performed in a 1:1 ratio with the HP CM2320fxi MFP scanner(Hewlett-Packard, Boise, ID, USA), and the SLA was considered as a reference due to its high accuracy. At the same time, the leaf area was determined by measurement (measured leaf area—MLA) based on descriptive parameters at the leaf lamina (Figure 2). Regarding the software analysis of the leaf surface, numerous research articles have promoted such methods due to the facilities they present primarily related to the precision and accuracy of the analyses [131,132,133]. The measured leaf area was determined based on the parameters MR × DV1, MR × DV2 and KA (KA_1_, KA_2_) surface constants determined for each cultivar, Relation (12), as well as individually, based on each parameter by regression analysis.

(12) MLA=MR ·DV·KA

where: *MLA*—measured leaf area; *MR*—mid rib; *DV*—can be: DV1—distance to the end of the venation VL1–VR1; DV2—distance to the end of the venation VL2–VR2; *KA*—can be: KA_1_—the corresponding surface constant DV1; KA_2_—the corresponding surface constant DV2.

### 4.5. Statistical Analysis

All data were analyzed using variance analysis (ANOVA) and regression analysis. The assessment of the measurement accuracy and prediction of the leaf area was made by calculating the minimum measurement error (ME) related to the scanned leaf area (SLA) considered as a reference and based on the R^2^ and RMSE parameters. Models were determined by regression analysis represented by polynomial functions of leaf surface prediction based on each determined leaf parameter. For statistical analysis of the results, the EXCEL application from the Microsoft Office 2007 package and the PAST software (University of Oslo, Norway) were used [134].

## 5. Conclusions

Surface constants (*KA*) were found for six vine cultivars and facilitated the determination of the measured leaf area (*MLA*) based on some foliar descriptor elements in conditions of high statistical safety (based on RMSE and ME). The elements on the left side of the median rib (VL1, VL2, DSL1 and DSL2) facilitated a more accurate prediction of the leaf area compared to those on the right (VR1, VR2, DSR1 and DSR2). Based on statistical safety parameters (R^2^, RMSE), we found that the descriptive elements on the left side of the leaves facilitated a higher accuracy in determining the leaf area compared to the homologous elements on the right side, which recommends their use for calculating the leaf area in the cultivars studied when using only one known descriptor element of the leaf. In the case of estimating the leaf area based on the angles α and β, no statistical certainty was registered, and the results were not taken into account. The equations obtained for determining the foliar surface are based on the foliar parameters of the leaves in the six cultivars of vines studied. They can be tested/used in other varieties from the same group of leaf typology as those studied, but they can be adapted to other varieties, taking into account the specific values of the foliar parameters. The proposed method has the advantage of providing multiple ways to determine the leaf area of the vine based on the geometry elements of the leaves taken into account. It can be tested and adapted to other plant species, with leaves similar in geometric typology, to the vine.

## Figures and Tables

**Table 1:** Values of leaf areas and venations sizes at the level of vines in the studied cultivars.

Cultivar	MR	VL1	VR1	DV1	VL2	VR2	DV2
CS	9.21 ± 0.20	7.54 ± 0.19	7.92 ± 0.15	10.82 ± 0.24	5.80 ± 0.14	5.94 ± 0.10	11.52 ± 0.23
MI	9.16 ± 0.59	7.98 ± 0.39	7.86 ± 0.48	12.64 ± 1.10	6.00 ± 0.26	5.82 ± 0.24	11.10 ± 0.33
MO	9.30 ± 0.78	7.78 ± 0.60	8.04 ± 0.54	12.86 ± 0.71	5.60 ± 0.47	5.78 ± 0.31	10.86 ± 0.76
Ch	11.30 ± 0.49	9.64 ± 0.51	10.06 ± 0.39	14.98 ± 0.61	7.22 ± 0.32	7.62 ± 0.43	14.44 ± 0.63
Vi	13.56 ± 0.21	11.76 ± 0.51	12.18 ± 0.46	16.42 ± 0.84	8.88 ± 0.53	8.48 ± 0.48	17.04 ± 0.78
MH	15.46 ± 0.61	13.94 ± 0.70	12.88 ± 0.74	21.00 ± 1.73	10.56 ± 0.75	10.10 ± 0.48	20.46 ± 1.02

Note. CS—‘Cabernet Sauvignon’; MI—‘Muscat Iantarnîi’; MO—‘Muscat Ottonel’; Ch—‘Chasselas’; Vi—‘Victoria’; MH—‘Muscat Hamburg’; MR—median rib; VL1—venation left of order I; VR1—venation right of order 1; DV1—distance at the end of the venations VL1—VR1; VL2—second-order venation left; VR2—second-order venation right; DV2—distance at the end of the venations VL2–VR2.

**Table 2:** Values of the parameters related to the lobes and angles of the leaf venations in the studied cultivars.

Cultivar	DSL1	DSR1	DSL2	DSR2	α	β
CS	3.69 ± 0.17	3.49 ± 0.09	3.52 ± 0.10	3.57 ± 0.12	44.46 ± 0.77	54.91 ± 1.56
MI	5.54 ± 0.32	5.00 ± 0.44	4.98 ± 0.16	4.84 ± 0.18	51.94 ± 1.47	49.43 ± 1.44
MO	4.74 ± 0.39	4.64 ± 0.48	4.28 ± 0.31	4.48 ± 0.27	57.98 ± 1.83	54.52 ± 1.24
Ch	3.48 ± 0.20	3.82 ± 0.29	3.58 ± 0.27	3.88 ± 0.28	57.17 ± 1.63	52.97 ± 1.57
Vi	9.34 ± 0.55	9.46 ± 0.48	7.68 ± 0.38	7.68 ± 0.44	41.91 ± 1.22	44.76 ± 1.31
MH	6.14 ± 0.73	6.04 ± 0.46	6.20 ± 0.59	6.36 ± 0.45	50.43 ± 1.21	51.44 ± 1.54

Note. CS—‘Cabernet Sauvignon’; MI—‘Muscat Iantarnîi’; MO—‘Muscat Ottonel’; Ch—‘Chasselas’; Vi—‘Victoria’; MH—‘Muscat Hamburg’; DSL1—sinus base distance 1 left to lamina base; DSR1—sinus base distance 1 right to lamina base; DSL2—sinus base distance 2 left to lamina base; DSR2—sinus base distance 2 right to lamina base; α—the angle between the median rib (MR) and the right venation of the first order (VR1); β—the angle between the first-order straight venation (VR1) and the second-order straight venation (VR2).

**Table 3:** Values of area constant (KA) depending on leaf area and statistic safety parameters in the studied vine cultivars.

	KA_1_					KA_2_				
	‘Cabernet Sauvignon’									
SLA (cm^2^)	97.53					97.53				
KA	0.95	0.96	0.97	0.98	0.99	0.90	0.91	0.92	0.93	0.94
MLA (cm^2^)	95.02	96.02	97.02	98.02	99.02	95.88	96.95	98.01	99.08	100.14
ME	−2.51	−1.51	−0.51	0.49	1.49	−1.65	−0.58	0.48	1.55	2.61
RMSE	4.9137	4.4959	4.2794	4.2948	4.5397	3.4282	3.0539	3.0338	3.3741	3.9835
	‘Chasselas’									
SLA (cm^2^)	154.07					154.07				
KA	0.89	0.90	0.91	0.92	0.93	0.91	0.92	0.93	0.94	0.95
MLA (cm^2^)	151.29	152.98	154.69	156.39	158.09	150.19	151.84	153.49	155.14	156.79
ME	−2.79	−1.09	0.61	2.31	4.01	−3.88	-2.23	−0.58	1.07	2.72
RMSE	18.0852	17.7521	17.5824	17.5809	17.7476	8.9994	8.2622	7.8268	7.7444	8.0259
	‘Muscat Hamburg’									
SLA (cm^2^)	302.83					302.83				
KA	0.90	0.91	0.92	0.93	0.94	0.93	0.94	0.95	0.96	0.97
MLA (cm^2^)	294.89	298.17	301.44	304.72	307.99	294.89	298.83	302.01	305.19	308.37
ME	−7.94	−4.67	−1.39	1.89	5.16	−7.18	−4.00	−0.82	2.36	5.54
RMSE	52.6654	52.3765	52.3025	53.1656	53.8714	41.2034	40.9519	40.9538	41.2089	41.7126
	‘Muscat Iantarnîi’									
SLA (cm^2^)	110.03					110.03				
KA	0.91	0.92	0.93	0.94	0.95	1.06	1.07	1.08	1.09	1.10
MLA (cm^2^)	107.58	108.76	109.95	111.13	112.31	108.14	109.16	110.18	111.19	112.11
ME	−2.45	−1.27	−0.08	1.10	2.28	−1.89	−0.87	0.15	1.17	2.19
RMSE	12.1725	12.0021	11.9556	12.4616	12.8615	16.8639	16.7988	16.7971	16.8588	16.9831
	‘Muscat Ottonel’									
SLA (cm^2^)	110.27					110.27				
KA	0.89	0.90	0.91	0.92	0.93	1.05	1.06	1.07	1.08	1.09
MLA (cm^2^)	108.21	109.42	110.64	111.85	113.07	108.46	109.49	110.53	111.56	112.59
ME	−2.06	−0.85	0.34	1.58	2.80	−1.81	−0.78	0.25	1.29	2.32
RMSE	8.8879	8.5431	8.3732	8.3887	8.5888	6.2165	6.0236	6.0199	6.2057	6.5649
	‘Victoria’									
SLA (cm^2^)	216.17					216.17				
KA	0.95	0.96	0.97	0.98	0.99	0.91	0.92	0.93	0.94	0.95
MLA (cm^2^)	211.59	213.82	216.05	218.28	220.50	210.63	212.95	215.26	217.58	219.89
ME	−4.57	−2.35	−0.12	2.11	4.33	−5.53	−3.22	−0.90	1.41	3.72
RMSE	13.8312	13.1502	12.8298	12.8971	13.3459	19.3721	18.8042	18.5139	18.5145	18.8057

Note. KA_1_—valid for MLA = MR × DV1 × KA_1_; KA_2_—valid for MLA = MR × DV2 × KA_2_; SLA—scanned leaf area (average leaf area for leaves, obtained by SLA method); MLA—measured leaf area; ME—minimal error, the lowest value is the best; RMSE—Root Mean Squared Error, the lowest value is the best. Area constant (KA) in each cultivar were considered depending on minimum values for ME and RMSE.

**Table 4:** Equations for predicted leaf area (*PLA*) based on foliar parameters and statistical accuracy parameters.

**Parameter *x***	Equation for Predicted Leaf Area (PLA)	Equation Number	Statistical Accuracy Parameters		
			R^2^	*p*	RMSE
MR	$PLA=1.2284x^{2}-0.1746x+1.035$	(1)	0.965	*p* << 0.001	19.7330
VL1	$PLA=0.3662x^{2}+22.575x-92.945$	(2)	0.988	*p* << 0.001	17.1495
VR1	$PLA=-0.5495x^{2}+40.855x-180.18$	(3)	0.739	*p* << 0.001	43.7254
DV1	$PLA=-0.1114x^{2}+22.493x-141.23$	(4)	0.846	*p* << 0.001	34.8094
VL2	PLA=37.68x−111.21	(5)	0.997	*p* << 0.001	22.9111
VR2	$PLA=1.489x^{2}+17.61x-47.077$	(6)	0.917	*p* << 0.001	32.4634
DV2	PLA=19.713x−115.45	(7)	0.959	*p* << 0.001	27.4804
DSL1	PLA=18.38x+64.325	(8)	0.485	*p* = 0.0103	70.1936
DSR1	PLA=18.231x+66.478	(9)	0.477	*p* = 0.0119	70.3778
DSL2	PLA=31.157x+7.935	(10)	0.806	*p* << 0.001	61.7832
DSR2	PLA=31.23x+4.4471	(11)	0.752	*p* << 0.001	63.2706

Note. PLA—predicted leaf area; MR—median rib; VL1—venation left of order I; VR1—venation right of order 1; DV1—distance at the end of the venations VL1—VR1; VL2—second-order venation left; VR2—second-order venation right; DV2—distance at the end of the venations VL2—VR2; DSL1—sinus base distance 1 left to lamina base; DSR1—sinus base distance 1 right to lamina base; DSL2—sinus base distance 2 left to lamina base; DSR2—sinus base distance 2 right to lamina base.

## Data Availability

Not applicable.
